# Functional roles of the amino terminal domain in determining biophysical properties of Cx50 gap junction channels

**DOI:** 10.3389/fphys.2013.00373

**Published:** 2013-12-18

**Authors:** Li Xin, Donglin Bai

**Affiliations:** Department of Physiology and Pharmacology, The University of Western OntarioLondon, ON, Canada

**Keywords:** gap junction channel, *V*_*j*_-gating, single channel conductance, dual whole cell patch-clamp, Cx50

## Abstract

Communication through gap junction channels is essential for synchronized and coordinated cellular activities. The gap junction channel pore size, its switch control for opening/closing, and the modulations by chemicals can be different depending on the connexin subtypes that compose the channel. Recent structural and functional studies provide compelling evidence that the amino terminal (NT) domains of several connexins line the pore of gap junction channels and play an important role in single channel conductance (γ_*j*_) and transjunctional voltage-dependent gating (*V*_*j*_-gating). This article reviews recent studies conducted on a series of mutations/chimeras in the NT domain of connexin50 (Cx50). Functional examination of the gap junction channels formed by these mutants/chimeras shows the net charge number at the NT domain to be an important factor in γ_*j*_ and in *V*_*j*_-gating. Furthermore, with an increase in the net negative charge at the NT domain, we observed an increase in the γ_*j*_ as well as changes in the parameters of the Boltzmann fit of the normalized steady-state conductance and *V*_*j*_ relationship. Our data are consistent with a structural model where the NT domain of Cx50 lines the gap junction pore and plays an important role in sensing *V*_*j*_ and in the subsequent conformational changes leading to gating, as well as in limiting the rate of ion permeation.

## Introduction

Gap junction channels provide a direct passage for ions and small signaling/metabolic molecules to be exchanged between neighboring cells. Each gap junction channel is formed by two hemichannels docked end-to-end at the extracellular domains. Each hemichannel is an oligomer of six connexins (Cxs). Connexins are a group of homologous proteins encoded from 21 genes in the human genome (20 genes in the rodent genome) (Sohl and Willecke, 2004). Each connexin has its own unique tissue distribution and each tissue cell often expresses one or more connexins (Simon and Goodenough, 1998; Laird, 2006).

Gap junction channels can be gated via a variety of factors, including pH changes, intracellular calcium concentrations and transjunctional voltage (*V*_*j*_) (Verselis et al., 1994; Bukauskas and Peracchia, 1997; Peracchia et al., 2000; Bukauskas and Verselis, 2004; Gonzalez et al., 2007). *V*_*j*_ is the voltage difference between the interiors of two gap junction linked cells. *V*_*j*_-dependent closure of the gap junction channel, known as *V*_*j*_-gating, is a common property for all characterized gap junction channels. However, the extent and sensitivity of *V*_*j*_-gating of different gap junction channels can vary drastically (Burt and Spray, 1988; Barrio et al., 1991; Verselis et al., 1994; Purnick et al., 2000). Earlier studies on Cx26, Cx32 and other connexins have indicated that among several domains, the NT domain and the residue within the NT domain play a crucial role in gating polarity, the properties of *V*_*j*_-gating and unitary conductance (Rubin et al., 1992; Verselis et al., 1994; Purnick et al., 2000; Musa et al., 2004; Peracchia and Peracchia, 2005; Srinivas et al., 2005; Dong et al., 2006; Gemel et al., 2006; Verselis and Srinivas, 2008; Kronengold et al., 2012). The molecular basis of *V*_*j*_-gating is not fully understood. Here we summarize some recent experimental evidence which shows that *V*_*j*_-gating properties and unitary conductance of Cx50 gap junction channels depend on the NT domain, especially, the charged residues. We also discuss our findings relating to the atomic structure of gap junction channels to examine the structure-function relationship of gap junction channels at the submolecular domains and individual amino acid residues.

## Structural models of gap junction channels

**Figure 1:**
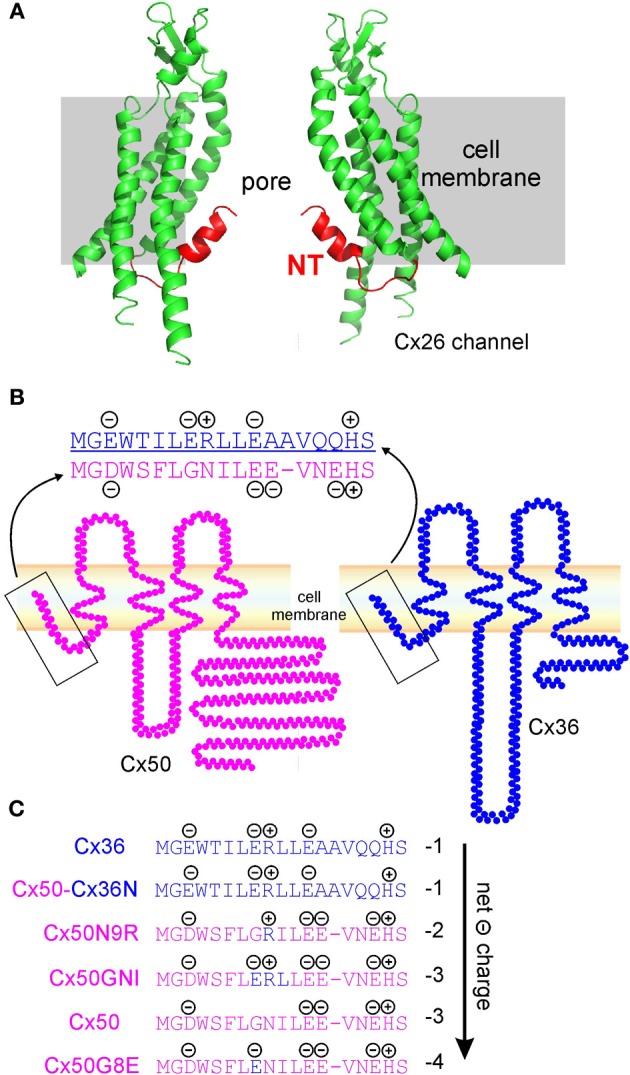
**Structure and topology models of Cx26, Cx50, and Cx36 gap junction channel. (A)** Crystal structure model of Cx26 channel. Only two subunits of a hemichannel are displayed according to the resolved part of Cx26 channel (PDB 2ZW3, Maeda et al., 2009). Amino terminal domain (NT, red) of each subunit folds into the gap junction channel and lines part of the pore surface. **(B)** Topology models of Cx50 (pink) and Cx36 (blue) and the sequence alignment of amino terminal domain (NT). Four negatively charged residues, and one positively charged residue, are found at the terminal portion of Cx50 NT, and three negatively charged, and two positively charged residues exist on Cx36 NT. **(C)** Net charges of the NTs of Cx36, Cx50, and Cx50 mutants (Cx50Cx36N, Cx50N9R, Cx50GNI, and Cx50G8E) were calculated and ranked from low to high negativity (arrow). Blue text depicts residues of Cx36 NT and pink text indicates the residues from Cx50 NT.

Gap junction channels are formed by the oligomerization of connexin molecules. Hydropathy analysis of connexin protein sequence predicts that each connexin has four transmembrane domains (M1–4), two extracellular domains (E1 and E2), one intracellular loop (IL), and the carboxyl terminus (CT) and the amino terminus (NT) in the cytosol (Nicholson, 2003; Saez et al., 2003). Based on various experimental approaches, several different gap junction channel structural models have been proposed and each provides some unique insights into the structure of the gap junction channel (Foote et al., 1998; Perkins et al., 1998; Unger et al., 1999; Muller et al., 2002; Nicholson, 2003). However, due to limited spatial resolution, the atomic structure of gap junction channels has not been resolved until recently. Maeda, Tsukihara and colleagues crystallized the Cx26 gap junction channel and provided the first atomic structure model at 3.5 Å resolution for this channel (Maeda et al., 2009; Suga et al., 2009). This high resolution structural model confirmed many structural features previously predicted. For example, the four transmembrane domains in each connexin form α-helical structures and the extracellular domains appear critical for the docking interactions (Maeda et al., 2009; Nakagawa et al., 2011; Gong et al., 2013). More importantly, the high resolution model provided greater detail and also revealed several novel structural features. One interesting, and somewhat surprising, feature of the Cx26 channel structure was that the NT domain of each connexin molecule folded into the gap junction pore and contributed to the lining of a portion of the pore inner surface (Figure 1A). In addition, six NT domains of Cx26 formed a funnel structure at the pore entrances and the funnels were the narrowest parts of the channel (Maeda et al., 2009; Maeda and Tsukihara, 2011). This kind of spatial arrangement provides a structural advantage for the NT domain in sensing a voltage change across the channel, which makes the NT domain a good determinant of *V*_*j*_-gating. The small physical channel pore diameter and the surface electrical charges at the site of NT domains form physical and electrical barriers to ions going through the channel, respectively. Therefore, the NT domain may also be important in limiting the rate of ion permeation through a channel, a parameter that can be quantitatively measured as the single (or unitary) channel conductance (γ_*j*_).

The NT domains, together with the transmembrane domains (M1–4), and extracellular loops (E1 and E2) of different connexins, are highly conserved among all connexins. We aligned the mouse Cx50 and Cx36 sequences with that of the human Cx26 using Jalview (Waterhouse et al., 2009). The results indicated that both Cx50 (56.4% sequence identity) and Cx36 (48.2% sequence identity) are highly similar to that of the Cx26 (Waterhouse et al., 2009). At this high level of conservation, it is reasonable to speculate that Cx50 and Cx36 may take a similar structure as Cx26 channels. We hypothesized that the NT domains for Cx50 and Cx36 line the respective gap junction channel pore and play an important role in their *V*_*j*_-gating and γ_*j*_. Topology structural models of Cx50 and Cx36 are shown in Figure 1B. We aligned the protein sequence of the beginning portion of the NT domains of Cx50 and Cx36 and labeled all the charged residues (Figure 1) (Xin et al., 2012a). It was very interesting to note that there are 5 charged residues in the NT of Cx36 (3 negative and 2 positive), and 5 charged residues in the NT of Cx50 (4 negative and 1 positive). A simple mathematical summation of all the charges gave the net charge of the Cx36 NT domain, −1, and that of the Cx50 NT domain, −3. Considering hexameric oligomerization of connexins to form one hemichannel, the net charge of the NT domains of the Cx36 hemichannel was −6 and that of Cx50 hemichannel was –18. End-to-end mirror symmetrical docking of two hemichannels was required to form a whole gap junction channel, which had twice the net charges of the NT domains. Thus, in theory, a minor change in the NT domain net charge is amplified 6 times in the hemichannel and 12 times in the whole gap junction channel. To test how the change in the NT net charge affects the channel *V*_*j*_-gating and γ_*j*_ properties, we systematically compared results across a number of mutants/chimera of Cx50.

## Functional studies using chimera and mutagenesis approaches

The technical approach we adopted was to heterologously express the mutant and wild-type connexins in gap junction deficient neuroblastoma (N2A) cells. Co-expression of the green fluorescent protein (GFP) was used to increase the success rate of identifying cell pairs expressing the mutant construct. We generated a series of mutants and chimeras, in which a single amino acid residue, or a short segment of amino acid residues between the two NT domains of the selected connexins, was exchanged. Using a double patch-clamp whole-cell recording method, we quantitatively measured *V*_*j*_-gating and γ_*j*_ of channels formed by these mutants/chimeras (Xin et al., 2010, 2012a,b). It was fortunate that many of these mutants/chimeras at the NT domain of Cx50 resulted in the formation of homotypic gap junction channels, with readily identifiable *V*_*j*_-gating characteristics and measurable γ_*j*_s. The generated chimera included Cx50Cx36N, in which the NT domain of Cx50 was replaced by that of the Cx36 (Figure 1C). Other mutants, Cx50N9R, Cx50GNI (a triple mutation composed of G8E, N9R and I10L), and Cx50G8E, were also generated with site-directed mutagenesis, using wild-type Cx50 as a template (Figure 1C).

We ranked the mutants, chimera and wild-type connexins according to the NT net charges in Figure 1C. The net charges range from −1 (Cx36 and Cx50Cx36N), −2 (Cx50N9R), −3 (Cx50GNI and Cx50) to −4 (Cx50G8E) in these mutants/chimera and wild-type connexins.

## *v*_*j*_-gating of Cx50, Cx36, and Cx50 mutants/chimera

Cx50 is expressed primarily in the lens and serves an important function in keeping the lens transparent (Saez et al., 2003). Knockout of the Cx50 gene in mouse leads to nuclear cataracts and microphthalmia (White et al., 1998). Cx36 is expressed in neurons and plays a role in synchronized neuronal activities (Perez Velazquez and Carlen, 2000). Mice lacking the Cx36 gene have disrupted or significantly reduced electrical coupling between interneurons in the neocortex and hippocampus, and reduced γ-frequency (30–70 Hz) rhythms in the hippocampus (Hormuzdi et al., 2001; Buhl et al., 2003). It is very hard to characterize gap junction channel properties in neurons due to one or more of the following reasons: extended arborization and complexity of neuronal dendritic trees, the existence of many active ion channels on the plasma membrane, and the remote location of gap junction channels from the recording sites (Connors and Long, 2004). The channel biophysical properties are mostly characterized from studies using recombinant expression of Cx36 or Cx50 in gap junction deficient cell lines (Srinivas et al., 1999b; Al-Ubaidi et al., 2000).

Homomeric homotypic gap junction channels formed by the Cx36 appear to be drastically different from the Cx50 channels in their *V*_*j*_-gating properties and single channel conductance (γ_*j*_). The gap junction channels formed by Cx36 possess one of the lowest single channel conductance (γ_*j*_ = 4–15 pS) among many characterized gap junction channels (30–300 pS) (Srinivas et al., 1999b; Teubner et al., 2000; Moreno et al., 2005). In addition, *V*_*j*_-gating of Cx36 gap junction channels are the weakest among all gap junction channels (more details are provided below). Cx50 gap junction channels, on the other hand, show large single channel conductance ~200 pS and pronounced *V*_*j*_-gating (Srinivas et al., 1999a; Bai et al., 2006; Xin et al., 2010). Little information is available as to which domains and amino acid residues of Cx36 or Cx50 are critical for their unique channel properties. We hypothesize that the *V*_*j*_-gating and single channel conductance (γ_*j*_) properties of the Cx36 or Cx50 gap junction channels are encoded in part by the NT domain.

**Figure 2:**
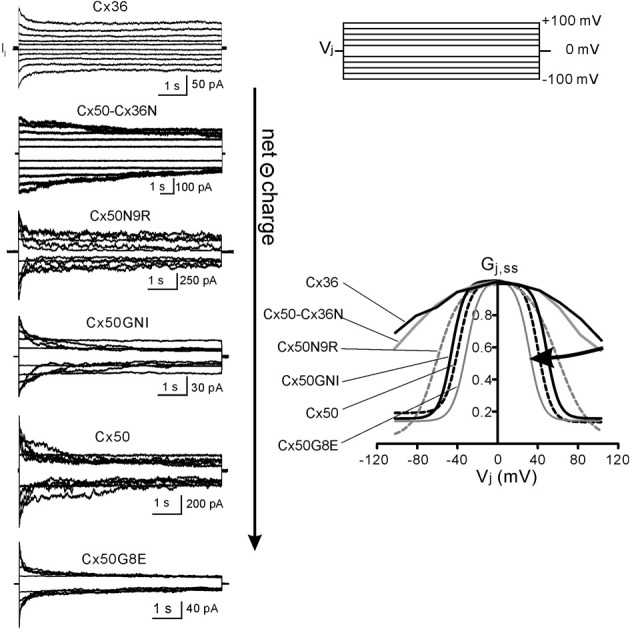
***V*_*j*_-gating of Cx36, Cx50, and the selected Cx50 NT mutants. Left** panel: superimposed junctional currents (*I*_*j*_*s*) in response to transjunctional voltage (*V*_*j*_) pulses (±20 to ±100 mV at 20 mV increment, shown on top **Right** panel) are illustrated. Each set of record was recorded from a pair of N2A cells expressing Cx36, Cx50-Cx36N, Cx50N9R, Cx50GNI, Cx50, and Cx50G8E as indicated. **Right** panel: steady-state junctional conductance, normalized to the peak junctional conductance (defined as *G*_*j,ss*_), was plotted as a function of *V*_*j*_. The *G*_*j,ss*_ − *V*_*j*_ plots of Cx36 and Cx50-Cx36N are directly from the experimental measurements, while the rest of the plots (smooth lines or dashed lines) are Boltzmann fittings from experimental data. With the increase of net charge negativity, the bell-shaped *G*_*j,ss*_ − *V*_*j*_ curves are getting narrower or with a lower values of *V*_0_ (half inactivation voltage in Boltzmann fitting). The data on Cx50, Cx36, Cx50-Cx36N, and Cx50N9R are from Xin et al. (2010) and the data on Cx50GNI and Cx50G8E are from Xin et al. (2012a) with permissions.

To investigate the role of the NT domain we generated a chimera (Cx50-Cx36N) in which the NT domain of the Cx36 was switched into Cx50. The *V*_*j*_-gating properties of Cx50-Cx36N displayed some novel properties and also adopted some features from their “parental” connexins. As shown in Figure 2, Cx50-Cx36N gap junction channels displayed non-inactivating junctional currents (*I*_*j*_*s*) in the *V*_*j*_*s* of ±20 to ±40 mV. When the *V*_*j*_*s* were in the range of ±60 to ±100 mV, a very slow *I*_*j*_ inactivation was observed, taking as long as 15 s to reach a steady-state. We calculated the coupling conductance (*G*_*j*_) at the steady state and at the initial peak of the *I*_*j*_*s*, and normalized the steady-state *G*_*j*_ to the peak *G*_*j*_ to obtain *G*_*j,ss*_. The *G*_*j,ss*_ − *V*_*j*_ relationship of Cx50-Cx36N was generated and was found to be very similar to that of Cx36 channels (Figure 2), indicating, as we anticipated that the NT domain is indeed very important in determining the *G*_*j,ss*_ − *V*_*j*_ relationship. We attempted to fit the data in the *G*_*j,ss*_ − *V*_*j*_ plots of both Cx36 and Cx50-Cx36N with the two-state Boltzmann equation, but the fittings could not converge. Thus, we only show the plot of *G*_*j,ss*_ − *V*_*j*_ on the averaged data for these two connexins.

To further identify the roles of the individual amino acid residues, especially the charged residues, we generated two single-point mutants (Cx50N9R and Cx50G8E) and a triple mutant (Cx50GNI). The net charge of the NT domain of these mutants was different, from −2 (Cx50N9R), −3 (Cx50GNI) to −4 (Cx50G8E) (see Figure 1C). Junctional currents (*I*_*j*_*s*) in response to *V*_*j*_*s* of ±20 to ±100 mV were recorded from N2A cell pairs expressing each of these mutants, and displayed together with those of wild-type Cx50 (the NT net charge is –3) (Figure 2, left panel). At *V*_*j*_*s* of ±20 mV, none of them showed much *I*_*j*_ inactivation. However, at *V*_*j*_*s* of ± 40 to ±60 mV, *I*_*j*_*s* of different mutants displayed different level of inactivation. In these mutants, the order of low to high level of inactivation of the *I*_*j*_*s* was Cx50N9R, Cx50GNI, Cx50, and Cx50G8E. At *V*_*j*_*s* of ±80 to ±100 mV, the inactivation of the *I*_*j*_*s* appeared to be constant in the following mutants: CX50GNI and Cx50G8E, and wild-type Cx50. Only Cx50N9R appeared to display further continued inactivation of I_*j*_s with the increased *V*_*j*_. The *G*_*j,ss*_ − *V*_*j*_ relationships in both positive and negative *V*_*j*_s of these mutants and wild-type Cx50 could be nicely fitted by the Boltzmann equation and were plotted together in Figure 2 (right panel) for comparison.

In the positive *V*_*j*_s, the *G*_*j,ss*_ was in a high conducting state in the low *V*_*j*_ range. With the increase of the *V*_*j*_, the *G*_*j,ss*_ became a much lower conducting state for each of the mutants. Mirror symmetrical *V*_*j*_-dependent changes on *G*_*j,ss*_ were observed in negative *V*_*j*_*s* for these mutants, so that the two Boltzmann fits for each of the mutants showed Y-axis symmetrical “bell”-shaped curves. A two-state Boltzmann model provided parameters describing the fitted curves for each polarity of *V*_*j*_. The definition of each Boltzmann parameters are listed as follows: *G*_max_ is the theoretical maximum gap junction conductance extrapolated from the experimental data; *G*_min_ is the extrapolated value of residual conductance; *V*_0_ is the half-inactivation voltage at which *G*_*j,ss*_ = (*G*_max_ + *G*_min_)/2 and *A* = *zq/KT* represents the voltage sensitivity (slope of the Boltzmann fitting curve) in terms of the number of equivalent gating charges, *z*, moving through the entire applied field, where *q* is the electron charge, and *K* and *T* are the Boltzmann constant and absolute temperature, respectively. One of these parameters, *V*_0_, clearly correlated with the increase of the net negative charge of the NT domain.

With the increase of the net negative charges from −2 to −4, the absolute values of *V*_0_ changed from 58–60 mV (Cx50N9R), 45–46 mV (Cx50GNI), 38–39 mV (Cx50) to 30–31 mV (Cx50G8E) (see Table 1 for details). Visually, the “bell” shaped Boltzmann fitted curves became narrower (Figure 2). Though our data on Cx36 and Cx50-Cx36N *G*_*j,ss*_ − *V*_*j*_ plots did not converge for Boltzmann fitting, previous studies on Cx36 channels showed a much higher *V*_0_ of 73–78 mV (Srinivas et al., 1999b) and of 85–87 mV (Moreno et al., 2005), indicating that the Cx36 channels, and possibly the Cx50-Cx36N channels, possess a higher *V*_0_. It is clear that with the increase in the net negative charge of the NT domain, the apparent *V*_0_ value is decreasing in these studied Cx mutants/chimeras, although the magnitude of the *V*_0_ change appears to be much more drastic with the net negative charge increase from −1 (Cx36 and Cx50-Cx36N) to −2 (Cx50N9R) than those *V*_0_ changes with the increases from −2 to higher levels of negative charges (Cx50GNI, Cx50, and Cx50G8E). *V*_0_ is a parameter describing the electrical field required to close 50% of the gap junction channels. A decrease in *V*_0_ can be achieved via several ways: a decrease in the stability of the open state, an increase of the stability of the closed/residue states, or the combination of both.

**Table 1:** **Boltzmann parameters and Gibb's free energy**.

	***z***	***V_0_* (mV)**	***G*_min_**	***ΔG*_*0*_ (kJmol^−1^**	***V*_j_**
Cx50N9R	2.3 ± 0.5	59.6 ± 2.7	0.04 ± 0.05	13.0 ± 3.1	−
	1.7 ± 0.3	57.8 ± 2.5	0.02 ± 0.05	9.4 ± 1.7	+
Cx50GNI	4.1 ± 0.7	45.2 ± 1.3	0.17 ± 0.02	18.2 ± 3.2	−
	4.2 ± 0.8	46.2 ± 1.5	0.17 ± 0.02	18.8 ± 3.6	+
Cx50	3.9 ± 1.0	38.4 ± 1.2	0.20 ± 0.01	14.6 ± 3.9	−
	4.0 ± 1.0	39.3 ± 1.0	0.15 ± 0.02	15.3 ± 4.0	+
Cx50G8E	3.8 ± 0.6	30.8 ± 1.8	0.15 ± 0.02	11.1 ± 1.9	−
	4.0 ± 0.7	30.2 ± 1.8	0.15 ± 0.02	11.5 ± 2.1	+

The values for V_0_ and ΔG_0_ are absolute values, where the “+” and “−” signs are ignored.

*Boltzmann equation:*
$$G_{j,\;ss}=\frac{\left(G_{\max}-G_{\min}\right)}{\left\{(1+\text{exp}\left[A(V_{j}-V_{0})\right]\right\}}+G_{\min}$$

Our experimental evidence indicates that the open dwell time is increased in Cx50-Cx36N, but decreased in Cx50N9R relative to that of wild-type Cx50 channels, which suggests that the open stability of the Cx50 gap junction channel is changed by these and possibly other studied mutants. More systematic studies are required to answer the question of whether the stability of the closed/residue states is also changed by these mutants.

## Unitary channel properties of Cx50, Cx36, and Cx50 mutants/chimera

Cx50 gap junction channels displayed a large single channel conductance (γ_*j*_ = 200 pS) at the main conducting state (also known as main state) and one subconducting state (also called substate or residue state) of approximately 40 pS (Srinivas et al., 1999a). Cx36 channel-mediated unitary channel conductance was much lower. Srinivas and colleagues reported a main γ_*j*_ of 10–15 pS (Srinivas et al., 1999b) and Moreno measured an even lower γ_*j*_ of 5 pS in pancreatic β-cells of Cx36 channels (Moreno et al., 2005). We tried to resolve the γ_*j*_ in our recording system and found that Cx36 channel unitary current was buried into the baseline noise level beyond the resolution of our recording setup, suggesting that the γ_*j*_ of Cx36 could be even lower. The consensus of the literature is that the main γ_*j*_ of Cx36 is very low and that of Cx50 is high, which begs the question: what will happen to the mutants/chimera when single or multiple amino acid residues of the Cx36 NT are switched into the Cx50? It was fortunate that all the mutants/chimeras listed in Figure 1C showed readily measurable unitary currents in poorly coupled cell pairs without any pharmacological interventions.

**Figure 3:**
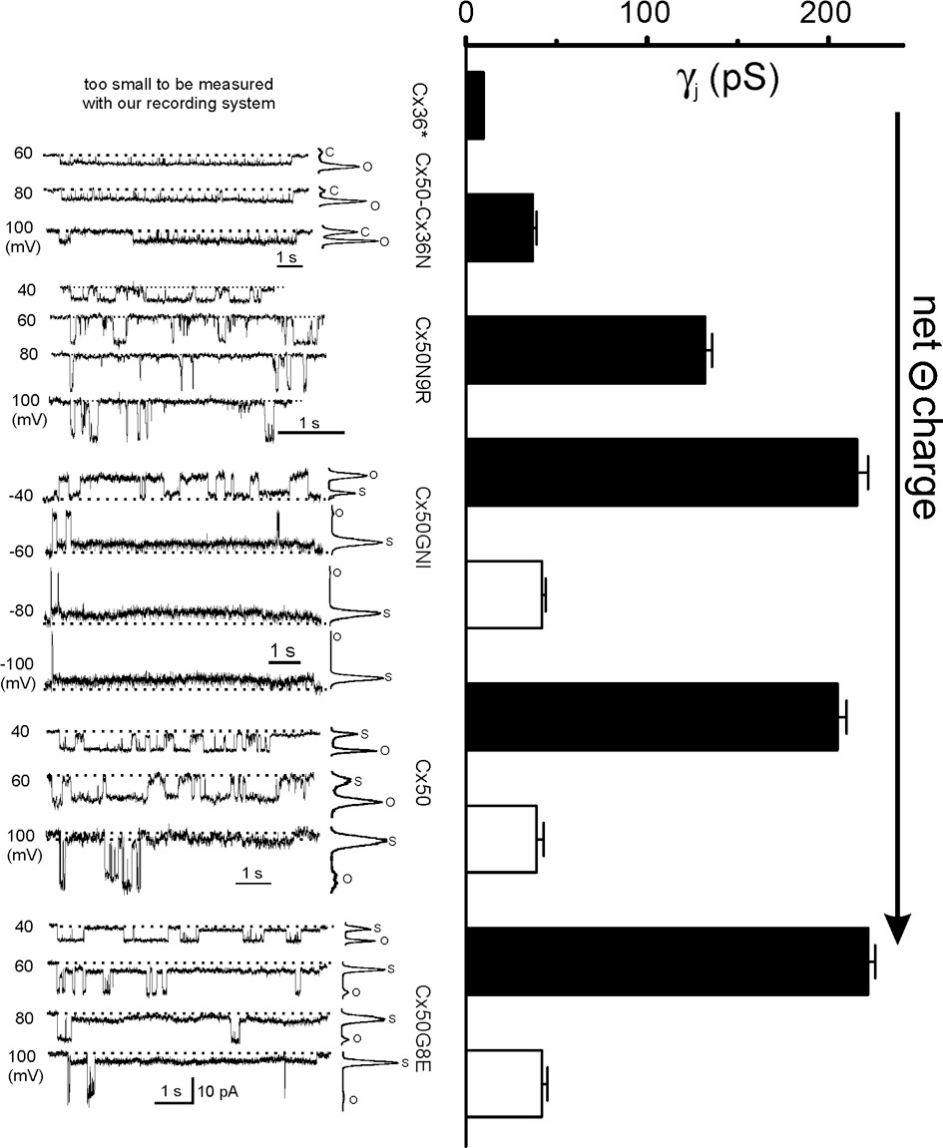
**Single channel conductance (γ_*j*_) of Cx36, Cx50, and Cx50 mutants**. Unitary currents recorded from N2A cell pairs expressing Cx36, Cx50-Cx36N, Cx50N8R, Cx50GNI, Cx50, and Cx50G8E are shown on the **Left**. On the **Right**, the bar graph summarized the averaged single channel conductance of the main open state (black bars) and the residue state (open bars). Data for Cx36^*^ gap junction channels are from (Srinivas et al., 1999b) and Moreno et al. (2005). The data on Cx50, Cx50-Cx36N, and Cx50N9R are from Xin et al. (2010) and the data on Cx50GNI and Cx50G8E are from Xin et al. (2012a) with permissions.

Cx50-Cx36N channel showed low level γ_*j*_ of around 40 pS (Figure 3). The conductance was almost identical to the substate conductance of the Cx50 channel, which made us wonder if the Cx50-Cx36N channel locked the channel in a subconducting state equivalent to that of the substate of Cx50 channel. However, our subsequent work on heterotypic Cx50/Cx50-Cx36N channels argued against this possibility. In the heterotypic channels of Cx50/Cx50-Cx36N, both main and subconductance states were evident (Xin et al., 2012b). Therefore, the reason for not being able to detect a clear substate for the homotypic Cx50-Cx36N channel must be a tiny conductance (beyond detection) combined with extremely low dwell time at the substate.

Homotypic Cx50N9R channels displayed a higher level of γ_*j*_ (132 ± 4 pS) than that of Cx50-Cx36N channels, and no long-lived subconductance states were observed in all the tested *V*_*j*_*s* (Figure 3). The gating transition time between the open state and the fully closed state (the average of 10–90% rise or decay time is 5.7 ms) was significantly longer than the transition time of the open state to the substate of the Cx50 channels (typical value of 10–90% rise or decay is ≤1 ms) (Xin et al., 2012b). However, it was also shorter than the transition time defining the slow *V*_*j*_-gating between the fully closed state to open/substate (~10 ms) of the Cx43 channels (Bukauskas and Peracchia, 1997).

The single channel currents recorded from the homotypic Cx50GNI and Cx50G8E channels were virtually identical to that of wild-type Cx50 channels. The main open state and subconductance state conductance of Cx50GNI (216 pS and 42 pS, respectively) and Cx50G8E (222 pS and 42 pS, respectively) were statistically the same as those of Cx50 channels (205 pS and 39 pS, respectively, Figure 3).

It is clear that the average of the main γ_*j*_ of the mutants/chimera increases with the increase of net negative charges at the NT domain. The γ_*j*_ range from below measurable level of Cx36 channels (with −1 at the NT domain), to a very low level of Cx50-Cx36N (−1) channels, an intermediate level of Cx50N9R (−2) channels to high level in Cx50GNI (−3), Cx50 (−3) and Cx50G8E (−4) channels. It appears that a saturation level for the main γ_*j*_ is reached when the net charge of the NT domain reaches −2 or lower. Our data are consistent with a structural model where the NT domain of Cx50 folds into, and lines, the initial portion of the gap junction pore. As the Cx50 channel is a cation preferred channel (Srinivas et al., 1999b), more negatively charged residues may help to attract more cations and increase the local cation concentration at the pore mouth (entrance), which would thus facilitate a higher rate of permeation through the gap junction channel. Conversely, reducing the net negative charge may alter the local charge density at the pore entrance and subsequently reduce the local cation concentrations. This may be the reason Cx50N9R and Cx50-Cx36N have a much lower γ_*j*_ than Cx50 channels. Interestingly, Cx50D3N also reduced the net charge of the NT domain and was found to decrease the single channel conductance of its hemichannels, which is consistent with our prediction (Peracchia and Peracchia, 2005; Srinivas et al., 2005).

## Final remarks

Here we discuss the importance of net negative charges at the NT domain for the *V*_*j*_-gating and γ_*j*_. Our experimental evidence shows a good inverse correlation of the net negative charge with the value of the Boltzmann parameter, *V*_0_. Additionally, a significant decrease of the net negative charge correlates with a decrease in the main γ_*j*_. We acknowledge, however, that in addition to charge changes, many other factors, including the size, polarization and hydrophobicity of the side chains of the amino acid residues in the NT, and other domains lining the pore, can also play a role in determining the biophysical properties of the gap junction channel. One example we encountered was the mutation Cx50D3E, where there was no change in the net negative charge, but only a minor change in the side chain size. This mutant greatly changed the *V*_*j*_-gating properties, with a substantial decrease of the gating charge (*z*), and the free energy between the open and closed states in the absence of the *V*_*j*_. This mutant was also shown to reduce the γ_*j*_ (Xin et al., 2012a). We believe that this third residue of the NT domain may be critical for maintenance of the normal structure of the pore, where a small change in the side chain of the amino acid residue could induce a big conformational change of the channel. Although we have learned a great deal in the past 30 years about gap junction channels, we are only just beginning to understand the detailed structure-function relationship of gap junction channels. New experimental and structural model approaches are needed to address those unresolved questions surrounding the specific gap junction channel properties.

### Conflict of interest statement

The authors declare that the research was conducted in the absence of any commercial or financial relationships that could be construed as a potential conflict of interest.

## Abbreviations

Cx: connexin
*G*_*j*_: gap junctional conductance
NT: amino terminus
γ_*j*_: single channel conductance
E1 and E2: extracellular domain 1 and 2, respectively
*V*_*j*_: transjunctional voltage.
